# Unfavourable beliefs about oral health and safety of dental care during pregnancy: a systematic review

**DOI:** 10.1186/s12903-023-03439-4

**Published:** 2023-10-15

**Authors:** Yasaman Mohammadi Kamalabadi, M. Karen Campbell, Natalie M. Zitoun, Abbas Jessani

**Affiliations:** 1 Department of Epidemiology and Biostatistics, Schulich School of Medicine & Dentistry, Western University, 1465 Richmond Street, London, ON N6G 2M1 Canada; 2 Department of Pediatrics, Schulich School of Medicine & Dentistry, Western University, 800 Commissioners Rd. E., London, ON Canada; 3 Department of Obstetrics & Gynecology, Schulich School of Medicine & Dentistry, Western University, 800 Commissioners Rd. E., London, ON Canada; 4 Children’s Health Research Institute, Lawson Health Research Institute, 750 Base Line Rd. E., London, ON N6C 2R5 Canada; 5 Department of Dentistry, Schulich School of Medicine & Dentistry, Western University, Dental Sciences Building, London, ON N6A 5C1 Canada

**Keywords:** Dental service utilization, Beliefs, Oral health, Pregnancy

## Abstract

**Background:**

Oral conditions such as gingivitis and periodontitis are correlated with adverse pregnancy outcomes such as preeclampsia, preterm birth and low birth weight. Oral health-related unfavourable beliefs can have negative influences on oral health behaviours including hesitation in accessing preventative dental treatments and dental service utilization. The objective of this systematic review was to examine unfavourable beliefs that expectant or new mothers frequently hold about oral health and the safety of dental care during pregnancy.

**Methods:**

An electronic database search on PubMed, Scopus, CINAHL, and MEDLINE (Ovid) followed by forward and backward citation tracing of the included studies was conducted. All English primary studies regardless of the year of publication were independently screened by two reviewers to identify studies addressing unfavourable beliefs about oral health and dental care during pregnancy. The CLARITY tool was applied to assess the risk of bias in the included studies.

**Results:**

Out of a total of 5766 records, 39 quantitative and six qualitative studies met the inclusion criteria. The commonly held unfavourable beliefs were regarding the safety of dental services utilization and dental treatment procedures, the adverse impacts of pregnancy on oral health, and oral hygiene necessity during pregnancy. The most discussed unfavourable beliefs included “pregnant women lose their teeth because of pregnancy” (*n* = 18), “dental treatments are not safe and harm the fetus” (*n* = 17), and “the developing baby absorbs calcium from the mother’s teeth” (*n* = 14).

**Conclusions:**

Unfavourable beliefs about oral health and dental care utilization are common among pregnant women and new mothers. The literature suggests that a low level of oral health knowledge and seeking information from social networks can contribute to such beliefs. This has implications for health promotion.

**Supplementary Information:**

The online version contains supplementary material available at 10.1186/s12903-023-03439-4.

## Background

Health beliefs refer to individuals’ perceptions and understanding of their own well-being, including their notions of health, factors contributing to their illness, and strategies to combat and recover from ailments [1]. In cases where there is a lack of concrete evidence and professional consensus, incorrect and unfavourable beliefs, if consistently reiterated, can be wrongly accepted as truths [2]. Health-related unfavourable beliefs, which can be due to a lack of scientific understanding, are common and can have negative influences on health and treatment-seeking behaviours [3–5]. Factors shown to be associated with a higher likelihood of believing health-related misinformation include lower education levels, lower healthcare knowledge, cultural customs and traditions, distrust in the healthcare system, and pre-existing false beliefs [6, 7]. Several health-related unfavourable beliefs associated with pregnancy have been perpetuated over centuries, some of which are still common, including some related to oral health especially during pregnancy [8, 9].

Oral health-related unfavourable beliefs are directly correlated with oral health service avoidance and higher unmet oral treatment needs of pregnant women [10]. Consequently, oral health conditions during pregnancy are considered “normal” and pregnant women are expected to cope with their oral conditions such as oral health-related pain, gingivitis, periodontitis, tooth decay and other dental conditions [11]. Further, unfavourable beliefs about the unsafety of dental treatment prevent pregnant women from dental visits [12]. Such unfavourable oral health beliefs that act as a barrier to oral health care services utilization can therefore leave oral conditions untreated which may result in decreased oral health-related quality of life and is correlated with adverse pregnancy outcomes such as preeclampsia, preterm birth and low birth weight [13, 14].

Given the adverse impacts of unfavourable beliefs on dental care services utilization and oral health status, it is important to identify such beliefs to dismantle false information and increase awareness of the importance of oral health during pregnancy. Therefore, this systematic review aimed to identify commonly held unfavourable beliefs about oral health and dental treatment during pregnancy.

## Methods

The protocol for this systematic review was registered with the International Prospective Register of Systematic Reviews, PROSPERO (ID: CRD42022358790) on September 19th, 2022. The Preferred Reporting Items for Systematic Reviews and Meta-Analyses (PRISMA) checklist was utilized as a guideline for the workflow of this systematic review [15]; details can be found in Additional file 1.

### Search strategy and study selection

We conducted an electronic search using four databases, PubMed, Scopus, CINAHL, and MEDLINE (Ovid) in May 2022. The search was updated in January 2023. We also conducted forward and backward citation tracing for included articles to locate relevant articles missed from this database search. The search terms were a combination of keywords and controlled vocabulary. The search strategy for all four databases is provided in Additional file 2.

The titles and abstracts of all articles identified through database searching were imported to COVIDENCE. It is a web-based systematic review program which facilitates most of the evidence synthesis steps including citation importing, screening, quality assessment, and data extraction [16]). Two reviewers (YMK and NMZ) independently performed the level 1 (title and abstract) screening. Subsequently, the full texts of the articles included after level 1 screening were uploaded to COVIDENCE for the purpose of full-text screening (level 2) which was independently conducted by the two reviewers based on the eligibility criteria (described below). Disagreements for both screening levels were solved through a discussion and reaching a consensus. For forward and backward citation tracing, all references and citing articles of the included studies were again added to COVIDENCE and the above screening steps were performed on them in the same way.

### Inclusion and exclusion criteria

The inclusion criteria were: 1) the study population was pregnant women or mothers of children under 6 years of age (as it is suggested that prenatal information can be accurately retrieved with a recall span of up to four to six years after delivery [17]); 2) the study was a primary investigation, regardless of study design and year of publication; 3) the article was written in English; 4) the study assessed beliefs about at least one of the impacts of pregnancy on a mother’s oral health; oral health on pregnancy; and dental treatment on pregnancy/child’s health. Further, because the focus of this search was unfavorable beliefs during pregnancy, a study was only included if it included statements referred to as such. The original labelling of such statements in the source manuscript may have been “misbeliefs”, “myths” and/or “false information”, but the labelling of the truth or false nature of such beliefs is largely subjective. Therefore, we have used the term “unfavorable beliefs” as a cumulative term throughout this paper.

The exclusion criteria included: 1) only assessed the knowledge of pregnant women regarding the child’s oral health and 2) assessed the beliefs or knowledge of dentists, midwives, gynecologists, and other health care providers.

### Data extraction and risk of bias assessment

Data were extracted by one reviewer (YMK) and then verified by a second reviewer (NMZ) using a standard form on Microsoft Excel (Version 16.70). The main collected data pertained to the studies’ characteristics including author, year of publication, country and region, study design, population, data collection tool, sample size, and response rate. Data on statements identified as misbeliefs and the prevalence of the participants believing in each statement were also extracted. Risk of bias in each study was assessed by utilizing five domains of the CLARITY tool, 1) representativeness of the source population, 2) adequacy of the response rate, 3) the proportion of missing data, 4) comprehensiveness, clarity, and face validity of the survey, and 5) reliability and construct validity of the survey [18].

### Data synthesis

Since the main purpose of this study was the identification of common unfavourable beliefs about oral health and dental service utilization during pregnancy, data were synthesized through narrative discussion. According to the objectives of this review and due to the wide variety of reported beliefs in the included studies, no meta-analysis could be undertaken. More importantly, the included studies were descriptive in nature and provided no further information than the range and/or the prevalence of the existing unfavourable beliefs among different populations of pregnant women.

## Results

**Fig. 1:**
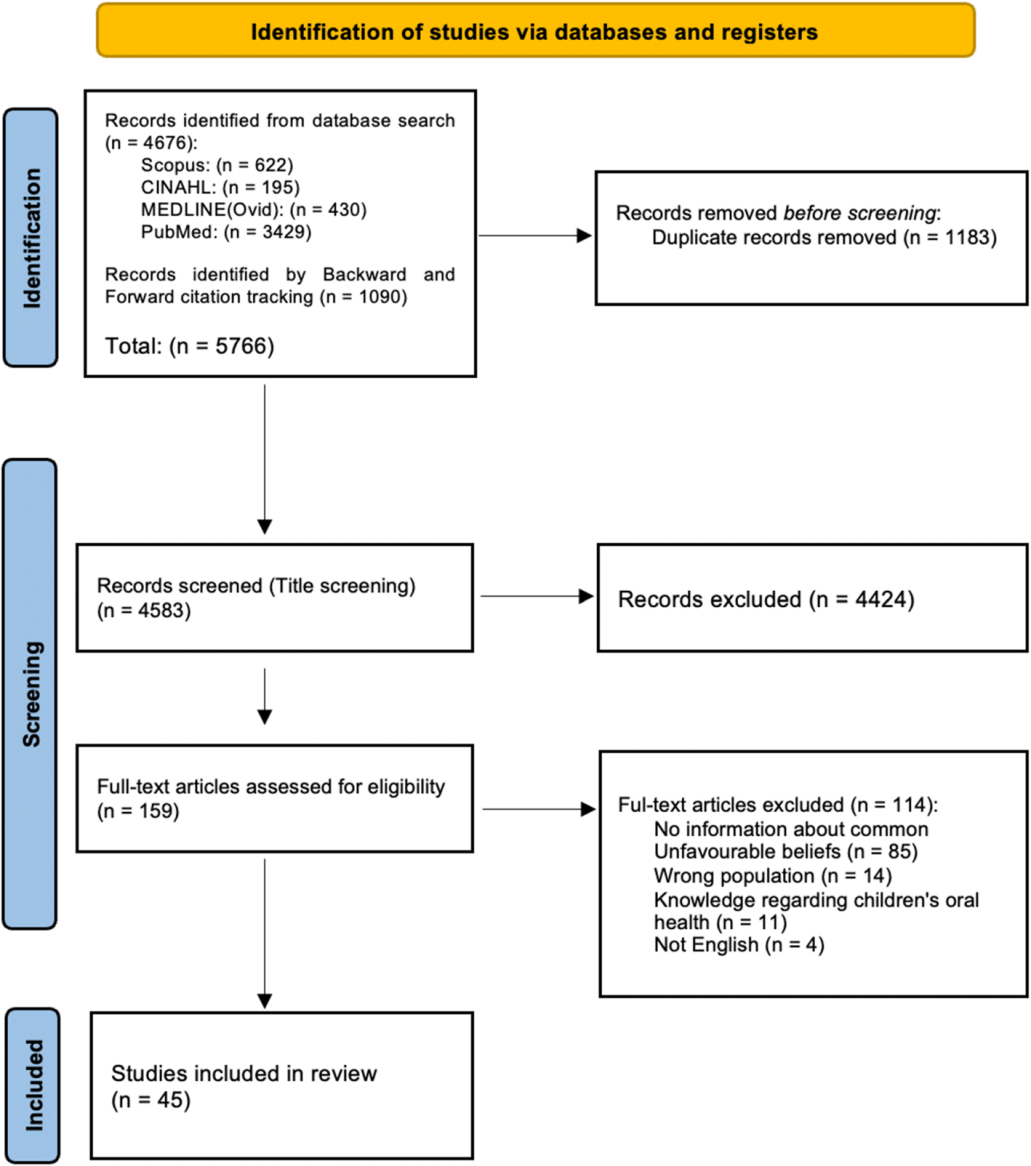
PRISMA diagram of study identification and selection

The flow diagram of study identification and selection is presented in Fig. 1. In total, 5766 studies were identified through database searching (*n* = 4676) and forward and backward citation tracing (*n* = 1090), out of which 1183 were removed due to duplication. After title screening was conducted, 159 studies remained. Full-text review of these and consideration of the inclusion and exclusion criteria resulted in 45 studies included in the review.

### Characteristics of the included studies

Table 1 summarizes the characteristics of the 45 studies which were included. All studies were cross-sectional in design. Sixteen studies used interviewer-administered questionnaires as a tool for data collection, two [11, 19] conducted semi-structured focus groups and interviews, and the other 29 studies gathered data using self-administered questionnaires. Six studies were qualitative [11, 19–23]. The oldest study was published in 1984 [24], and the newest ones were published in 2022 [25–29]. Sample sizes ranged from 19 in a qualitative study [21] to 801 in a quantitative study [29].

**Table 1:** Summary of the studies which met the inclusion criteria to investigate common oral health-related unfavourable beliefs during pregnancy

Citation	Author (year)	- Country - Region/City	Study design	Data collection tool	Sampling method	Sample size (N)	Response rate (%)	Theme of the manuscript^a^
[24]	Jago et al. (1984)	- Australia - Brisbane	Cross-sectional	Self-administered questionnaire	Consecutive sampling	441	98%	Oral health attitudes
[30]	Al Habashneh et al. (2005)	- USA - Johnson County, Iowa	Cross-sectional	Self-administered questionnaire	No information	625	69%	Dental services utilization – Oral health knowledge
[31]	Dinas et al. (2007)	- Greece - Thessaloniki	Cross-sectional	Self-administered questionnaire	No information	425	90%	Dental services utilization
[32]	Saddki et al. (2010)	- Malaysia - Kelantan	- Cross-sectional	Self-administered questionnaire	Systematic random sampling	124	100%	Dental services utilization
[23]	Detman et al. (2010)	- USA - Florida	Cross-sectional (qualitative)	Interview + pre-existing dataset	No information	253	47%	Oral health beliefs
[33]	Boggess et al. (2011)	- USA - North Carolina	Cross-sectional	Self-administered questionnaire	No information	599	88%	Oral health knowledge and beliefs
[34]	Abiola et al. (2011)	- Nigeria - Ikeja	Cross-sectional	Self-administered questionnaire	Consecutive sampling	453	No information	Oral health knowledge and attitudes
[35]	Hashim (2012)	- United Arab Emirates - Dubai, Sharjah and Ajman	Cross-sectional	Self-administered questionnaire	Computer-generated random sampling	750	94%	Oral health knowledge—Dental services utilization
[36]	Özen et al. (2012)	- Turkey - Ankara, Gülhane Medical Academy’s Department	Cross-sectional	Interviewer-administered questionnaire	No information	351	93%	Oral health knowledge
[22]	Murphey (2013)	- USA - Southwestern United States	Cross-sectional (qualitative)	Interview	Convenience, purposive sampling	24	No information	Myth about oral health
[37]	George et al. (2013)	- Australia - Southwestern Sydney	Cross-sectional	Self-administered questionnaire	Convenience sampling	241	77%	Oral health knowledge
[38]	Amin et al. (2014)	- Canada - Edmonton	Cross-sectional	Self-administered questionnaire	Convenience sampling	423	100%	Myths about oral health—Dental service utilization
[39]	Gupta et al. (2015)	- India - Chandigarh	Cross-sectional	Self-administered questionnaire	Computer-generated random sampling	200 pregnant and 200 non pregnant women	No information	Oral health knowledge and attitude
[40]	Sajjan et al. (2015)	- India - Bagalkot District	Cross-sectional	Interviewer-administered questionnaire	Convenience sampling	332	No information	Oral health knowledge
[41]	Przeklasa et al. (2015)	- Poland - Cracow, Myslenice and Mszana Dolna	Cross-sectional	Questionnaire	No information	132	No information	Oral health knowledge
[42]	Assery (2016)	- Saudi Arabia - Al-Jubail	Cross-sectional	Self-administered questionnaire	No information	252	84%	Oral health knowledge
[43]	Nagi et al. (2016)	- India - Balispur city	Cross-sectional	Interviewer-administered questionnaire	Convenience sampling	446	No information	Oral health knowledge
[44]	Gaffar et al. (2016)	- Saudi Arabia - Dammam	Cross-sectional	Self-administered questionnaire	Consecutive sampling	197	91%	Oral health knowledge
[21]	Al Khamis et al. (2016)	- Kuwait - Al Asimah, Hawalli, Al Ahmadi, Al Farwaniyah, Al Jahra	Cross-sectional (qualitative)	Interview	Purposive sampling	19	53%	Oral health misbeliefs, knowledge, and attitudes
[45]	Gonik et al. (2017)	- USA - Detroit	Cross-sectional	Self-administered questionnaire	No information	Urban: 208 and—suburban: 54	100%	Oral health knowledge and behaviour
[46]	Abdalla et al. (2017)	- Egypt - Minia Governorate	Cross-sectional	Interviewer-administered questionnaire	Purposive sampling	300	No information	Oral health attitudes and knowledge
[47]	Abel-Aziz et al. (2017)	- Egypt - El-Fayoum	Cross-sectional	Interviewer-administered questionnaire	Convenience sampling	266	No information	Oral health beliefs
[48]	Khalaf et al. (2018)	- Egypt - Assiut Governorate	Cross-sectional	Interviewer-administered questionnaire	Systematic sampling	365	No information	Oral health attitudes and knowledge
[49]	Barbieri et al. (2018)	- Brazil - Southern region of the city of São Paulo	Cross-sectional	Self-administered questionnaire + prenatal records	Convenience sampling	195	86%	Oral health knowledge
[50]	Abu-Hammad et al. (2018)	- Saudi Arabia - Al Madinah	Cross-sectional	Self-administered questionnaire	Convenience sampling	360	No information	Oral health knowledge and beliefs
[11]	Bahramian et al. (2018)	- Iran - Tehran	Cross-sectional (qualitative)	Semi-structured in-depth interviews (IDIs) and focus group discussions (FGDs)	No information	22	74%	Dental services utilization—Oral health knowledge and misbeliefs
[51]	Ishaq et al. (2018)	- Pakistan - Lahore	Cross-sectional	Self-administered questionnaire	No information	121	No information	Oral health knowledge and attitudes
[19]	Lubon et al. (2018)	- Nepal - Rural Sarlahi district	Cross-sectional (qualitative)	Semi-structured in-depth interviews (IDIs) and focus group discussions (FGDs)	Purposive sampling	Interviews (*n* = 16) and focus group discussions (3 groups, *n* = 23)	No information	Dental services utilization—Oral health knowledge and attitudes
[52]	Gupta et al. (2019)	- Nepal - Biratnagar	Cross-sectional	Interview	Convenience sampling	50	No information	Oral health knowledge
[53]	Hans et al. (2019)	- India - No information	Cross-sectional	Self-administered questionnaire	No information	225	No information	Oral health knowledge
[20]	Liu et al. (2019)	- China - Hong Kong, Tsan Yuk Hospital	Cross-sectional (qualitative)	Semi-structured interview	No information	30	75%	Dental services utilization – Oral health-related information acquisition
[54]	Rafeek et al. (2019)	- Jamaica - Trinidad	Cross-sectional	Self-administered questionnaire and oral health examination	Convenience sampling	161	No information	Oral health knowledge
[55]	Barman et al. (2019)	- India - Khurda district, Bhubaneswar	- Cross-sectional - -	Self-administered questionnaire	No information	300	No information	Dental services utilization
[56]	Llena et al. (2019)	- Spain - Comunidad Valenciana	Cross-sectional	Self-administered questionnaire	Random sampling	139	100%	Oral health knowledge
[57]	Kamaruddin et al. (2019)	- Malaysia - Hospital Universiti Sains Malaysia, Kelantan	Cross-sectional	Self-administered questionnaire	Convenience sampling	76	95%	Oral health knowledge
[58]	Awasthi et al. (2020)	- Nepal - Lalitpur	Cross-sectional	Interview	Purposive sampling	114	No information	Oral health attitudes
[59]	Soegyanto et al. (2020)	- Indonesia - Central Jakarta	Cross-sectional	Self-administered questionnaire	No information	167	No information	Oral health knowledge
[60]	Riaz et al. (2020)	- Pakistan - Rawalpindi	Cross-sectional	Interviewer-administered questionnaires	Convenience sampling	260	No information	Oral health knowledge and attitudes
[61]	Chinenye-Julius et al. (2021)	- Nigeria - Ijebu, Ogun State	Cross-sectional	Self-administered questionnaire	Convenience sampling	385	No information	Oral health knowledge and attitudes
[12]	Jain et al. (2021)	- India - Karnal, Haryana	Cross-sectional	Self-administered questionnaire	No information	380	No information	Myths about oral health
[25]	Azarshahri et al. (2022)	- USA - Facebook	Cross-sectional	Self-administered online questionnaire	Purposive and snowball sampling	622	No information	Myths about oral health—Dental services utilization
[26]	Gavic et al. (2022)	- Republic of Croatia - Social platform Facebook	Cross‐sectional	Self-administered online questionnaire	Volunteer sampling	325	No information	Oral health attitudes and knowledge
[27]	Javali et al. (2022)	- India - Deccan, South India	Cross-sectional	Self-administered questionnaire	Random sampling	445	92%	Oral health knowledge and attitude
[28]	Kaba et al. (2022)	- Kenya - Western part of Kenya	Cross-sectional	Interviewer-administered questionnaire	Systematic sampling	309	100%	Oral health knowledge, attitude, and barriers
[29]	Akbari et al. (2022)	- Indonesia - No information	Cross-sectional	Self-administered online questionnaire	No information	801	No information	Myths about oral health

^a^Unfavourable beliefs were identified and extracted from the following extracted manuscripts

### Risk of bias assessment

**Fig. 2:**
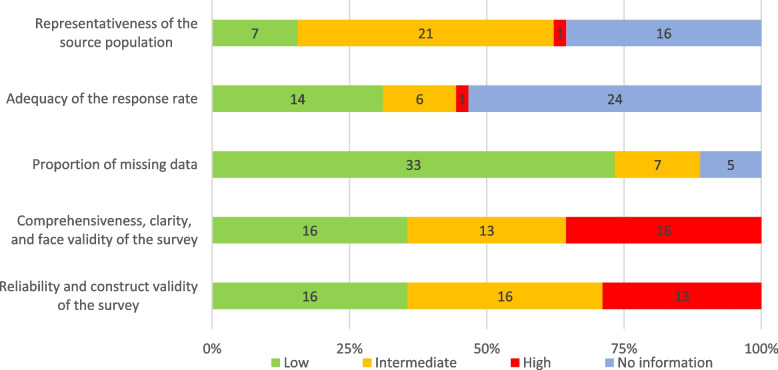
Summary of risk of bias assessment of the included studies using the CLARITY tool

Figure 2 shows the summary of findings from the risk of bias (RoB) assessment of the included studies. Only two studies [35, 56] had a low RoB in all five domains. A domain was marked as “No information” for a study if the related information was not exactly provided by the authors. The “probably yes” and “probably no” answers were combined and marked as “Intermediate” risk of bias. The “comprehensiveness, clarity, and face validity of the survey” and “reliability and construct validity of the survey” were the most common risks of bias with 16 (36%) and 13 (29%) of the studies having a high RoB in these domains, respectively. 16 (36%) studies did not provide information about the sampling method 21 (47%) studies recruited their samples through non-random (consecutive, convenience, and purposive) sampling, resulting in an intermediate risk of bias. Almost half (53%) of the studies did not report the response rate; however, this rate was more than 75% in 14 (31%) of the studies. Regarding missing data, 33 (73%) of the studies had a low risk of bias. A detailed RoB assessment of each study is presented in Additional file 3.

More than one hundred uniquely worded unfavourable beliefs were identified. However, many of these are equivalent and simply differently worded. These words can be grouped into four main categories of unfavourable beliefs about: 1) utilization of dental services (Table 2); 2) specific dental treatment procedures (Table 3); 3) oral health conditions (Table 4); and 4) other oral health-related unfavourable beliefs (Table 5). Due to the variety of how different studies worded their questions and labelled statements (as knowledge, attitude, misbelief, perception, myths, view, and misconception), for consistency purposes, the word “unfavourable beliefs” is used throughout this review. The original statements and their prevalence can be found in Additional file 4.

**Table 2:** Unfavourable beliefs about utilization of dental services during pregnancy

**Unfavourable beliefs about dental treatment**	• Dental treatment adversely affects the fetus / is not safe [21, 22, 25, 28, 31, 32, 37–39, 42, 44, 47, 51, 52, 54, 55, 57]. • Dental treatment should be avoided during pregnancy (reasons not specified) [28, 47, 49]. • Teeth may not be treated during pregnancy (reasons not specified) [23, 36, 41, 59]. • Pregnant women should seek dental treatment only in case of emergency [37, 49, 53, 54, 57, 60].
**Unfavourable beliefs about dental visit**	• Dental visits are not necessary [11, 34, 41, 47, 58, 61]. • Dental visits are not safe [39, 44, 53, 61]. • Dental visits should be avoided (reasons not specified) [12, 20, 29, 30]. • Dental visits for a check-up are not safe [28, 33, 48, 50].

**Table 3:** Unfavourable beliefs about the impact of specific dental treatment procedures on pregnancy

**Dental procedures**	**Unfavourable beliefs**
**X-rays/exposure to radiation**	• It is harmful to the fetus and/or mother [22, 26–28, 40, 42, 43, 45, 50, 51]. • It causes miscarriage [11]. • It is completely contraindicated [56].
**Local anesthesia**	• It is not safe for pregnant women and/or the fetus [26, 42, 45]. • It affects the development of the baby’s organs [12, 29]. • It is contradicted during pregnancy [41, 56]. • It causes miscarriage [11].
**Medication**	• It should be avoided during pregnancy [20]. • Medication prescribed by dentists may harm the fetus or mother [11, 27, 45].
**Dental extraction**	• It causes miscarriage [12, 29]. • It adversely affects the fetus [42]. • It should not be done during pregnancy/is not safe [23, 26, 28, 51].
**Other**	• Dental filling/crown is not safe [26, 42, 45]. • Calculus removal/ scaling/ tooth cleaning is not safe [26, 28, 42, 45]. • Periodontal treatment is not safe [26]. • Dental environment (material, noise) is harmful to the baby [20, 22].

**Table 4:** Unfavourable beliefs about the impact of pregnancy on oral health

**Oral conditions**	**Unfavourable beliefs**
**Tooth loss**	• A tooth per child [11, 21, 24, 30, 35, 36, 59]. • Pregnancy causes tooth loss/ Pregnant women can lose teeth only because of pregnancy [28, 33, 39, 43, 46, 48, 50, 58]. • It is normal to lose a tooth during pregnancy [25, 38, 45].
**Periodontal/gingival problems**	• Pregnancy causes periodontal problems [24, 34, 42, 53, 58]. • Gum bleeding/swollen gums/pain in gums is normal [12, 28, 29, 39, 45, 53].
**Tooth decay**	• Pregnancy causes tooth decay [24, 42]. • Dental decay is normal in pregnancy [45].
**Other**	• Dental pain is normal [20, 21, 45]. • Poor oral health is normal during pregnancy [25, 38].

**Table 5:** Other oral health-related unfavourable beliefs during pregnancy

**Unfavourable beliefs about calcium**	• Dental caries/tooth loss is due to the loss of calcium [48, 49]. • Calcium is drawn out of a pregnant woman’s teeth during pregnancy [11, 20–23, 25, 29, 30, 36, 38, 42, 46, 48, 59]. • Lost calcium can be compensated by swishing and keeping milk in the mouth [22]. • Calcium supplements suffice for dental problem prevention [11, 20]. • Calcium consumption during pregnancy stimulates primary teeth eruption [29].
**Other unfavourable beliefs**	• Teeth should not be brushed after delivery (for a while) [12, 29, 39]. • Teeth should not be brushed during pregnancy [12, 29]. • Uncomfortable feeling in teeth is because of “yeet hay” (internal heat) [20]. • The mother’s diet during pregnancy affects the child's teeth color [22]. • Poor oral hygiene causes cavities that enter the abdomen and negatively affect the baby [19].

#### Unfavourable beliefs about utilization of dental services

Table 2 presents unfavourable beliefs around two domains: that dental treatment is unsafe or should be avoided; or that dental visits should be avoided during pregnancy due to unsafety or unnecessity. Gupta et al. [39] reported these as two distinct beliefs; therefore, all other statements about these two domains are separately shown. These were discussed in 35 studies, for which 12 indicated that the belief that dental treatment was unsafe is one of the barriers to dental utilization [25, 30–32, 36–38, 52, 54, 55, 57, 59].

#### Unfavourable beliefs about specific dental treatment procedures

Table 3 presents unfavourable beliefs about specific dental treatment procedures.

### X-rays and exposure to radiation

Twelve studies reported concerns about radiation exposure in dental clinics. The study by Murphey [22] identified a belief regarding the harmfulness of dental X-rays to the fetus, which was considered to be a false belief. Similarly, a qualitative study by Bahramian and colleagues [11] identified the belief that radiography could cause miscarriage; the authors of the study considered it to be a “misbelief”. Other studies cited in Table 3 reported the prevalence of unfavourable beliefs regarding adverse pregnancy outcomes due to dental X-rays exposure which ranged from 31% [50] to 93% [27].

### Dental anesthesia and medication

Eight studies reported the unfavorable belief among pregnant women that dental anesthesia was hazardous, with the prevalence of this belief ranging from 25% [12] to a high of 95% [45] of participants in different studies. In a study by Bahramian et al. [11], an interviewee believed that anesthesia causes miscarriage. In four studies, participants were reported to believe that medications pertinent to dental treatments, such as pain medications, should be avoided because of harm to the fetus or mother [11, 20, 27, 45].

### Dental extraction

Seven studies discussed dental extraction during pregnancy. One of the common beliefs identified by Jain et al. [12] and Akbari et al. [29] was that tooth extraction causes miscarriage. In one qualitative study [23], and four other quantitative studies, pregnant women believed that dental extraction is contraindicated and harmful. When reported, the prevalence of the belief that extraction is a harmful dental practice ranged from 6% in a study by Assery [42] to more than 80% of the respondents in three other studies [26, 28, 51].

### Other dental treatments

Routine dental treatment procedures such as dental fillings, calculus removal, and periodontal therapy were perceived to be unsafe by participants in four quantitative studies [26, 28, 42, 45]. In qualitative studies, concerns about bacterial infection of the baby due to bleeding from dental check-ups were also stated. Some participants expressed potential environmental sources of harm including dental clinics exposing their fetuses to bacteria, materials and chemicals used in dental offices [20, 22].

#### Unfavourable beliefs about oral conditions during pregnancy

Table 4 presents unfavourable beliefs regarding the impact of pregnancy on oral health.

### Tooth loss

Eighteen studies discussed tooth loss and pregnancy. In five studies participants were specifically asked if they held unfavourable belief in the given statement/s “a tooth per child” [24, 30, 35, 36, 59] while in two other qualitative studies, participants indicated an unfavourable belief that “at least one tooth is lost or destroyed in each pregnancy” [11, 21]. This belief was assessed through various other statements as shown in Table 4. The prevalence of beliefs about tooth loss were notably different; in a study by Kaba et al. in Kenya [28] 86% of participants unfavourably believed that pregnancy causes tooth loss while in the study by Awasthi et al. [58], no one attested to this belief.

### Periodontal and gingival problems

As shown in Table 4, in 10 studies, participants believed that pain in gums, bleeding or swollen gums are normal occurrences and need no special consideration [12, 28, 29, 39, 45, 53], or that pregnancy is a cause of periodontal problems [24, 34, 42, 53, 58].

### Other oral conditions

Although not so common, some women perceived toothache [20, 21, 45] or tooth decay as normal occurrences in pregnancy [24, 42, 45].

#### Other unfavourable beliefs

One of the most commonly held beliefs during pregnancy was that calcium is absorbed from the teeth by the developing fetus. At least 37% of the participants had this perception in the study of Assery [42] and the study of Abdalla et al. [46] had the highest proportion of participants with this belief (80%). One qualitative study attributed the uncomfortable tooth sensation to “yeet hay” (internal heat) which could be solved by consuming herbal tea [20]. Another belief reported by adolescent pregnant mothers in the US was that the colour of the food in mothers’ diets is associated with the appearance of their babies’ teeth. They believed that eating white ice cream during pregnancy can cause white teeth in the baby and having beans and meat in the diet can cause an unhealthy appearance of the teeth [22].

Three studies showed that pregnant women believed that teeth should not be brushed during pregnancy or after delivery (from a few days to a few weeks) [12, 29, 39]. The reasons they did not brush their teeth either during or right after pregnancy were that they were not allowed by family members, or it is not good for the baby’s health, or it makes the teeth loose [12]. Other myths and misconceptions reported in the included studies are shown in Table 5.

## Discussion

Our study aimed to identify commonly held unfavourable beliefs about oral health and dental treatment during pregnancy. Understanding unfavourable beliefs about oral health and dental treatment, as well as understanding factors associated with those beliefs, is important to create risk-based preventative intervention and oral health awareness during pregnancy. This systematic review revealed unfavourable beliefs about the association of pregnancy with oral health, the safety of dental care, different dental treatment, and oral hygiene practices. This discussion will summarize those findings within the context of the potential consequences of unfavourable beliefs and oral health-related information resources.

Out of a total of 45 included, the majority of the studies reported unfavourable beliefs about the utilization of dental care during pregnancy, irrespective of the type of dental treatment. In two studies more than half of the participants believed that visiting a dentist even for regular check-ups is not safe and should be avoided during pregnancy [48, 50]. Evidence suggests that such beliefs can prevent pregnant women from seeking timely dental treatment during pregnancy which can impact the overall well-being and oral health-related quality of life of pregnant women [31].

Our review identified unfavourable beliefs regarding the safety of specific dental procedures such as the use of local anesthetics, medication prescription, dental radiography, restorative procedures, and periodontal treatment. Women’s concerns regarding dental radiographs were the most discussed dental procedure in the included studies. This belief can be due to a lack of awareness about the safety of dental radiography among pregnant women and their healthcare providers [62]. With low doses of radiation (ALARA as low as reasonably achievable) [63, 64] and additional safety measures such as lead aprons and thyroid collars, dental radiographs are considered safe for all stages during pregnancy [65]. Not being adequately informed about the safety of dental treatments, pregnant women may choose to postpone receiving dental care until after delivery without knowing the adverse effects of untreated oral conditions such as odontogenic infection [66].

In the included studies, unfavourable beliefs about oral conditions during pregnancy were also widely held, with the old saying “a tooth per child” being one of the most prevalent ones. Although there is no causal link between parity and tooth loss [67, 68], some studies on this association showed that women with more children had more missing teeth [69–72]. Russell et al. concluded that the cumulative destructive impact of untreated periodontal diseases due to lack of access to preventative periodontal care can contribute to tooth loss and may explain this association [70].

Our results highlighted that gum diseases and dental caries were considered ‘normal’ during pregnancy and that pregnancy was believed to ‘cause’ these oral diseases. One possible explanation for these beliefs is that pregnant women may experience periodontal problems due to hormonal changes [73–75], frequent nausea and vomiting, and altered oral hygiene and snacking behaviours which makes them susceptible to oral conditions such as tooth decay and gingival conditions [76]. Experiences of oral conditions during previous pregnancies may also lead to such beliefs. For example, those who experienced tooth cavities in their previous pregnancy stated that it is ‘normal that at least one tooth be destructed in this period’ [11]. Therefore, it is recommended by the American Dental Association that pregnant women should seek preventative dental care during pregnancy [77].

In almost one-third of the included studies, the belief that calcium is absorbed from teeth by the fetus’s development and that calcium loss results in tooth problems were reported; however, there is no evidence to support such beliefs. A study on mineral concentrations of extracted teeth showed no difference between calcium concentrations of pregnant and non-pregnant women’s teeth [78]. In fact, the higher dental caries and tooth loss experience may relate to physiological alterations and behavioural changes such as frequent snacking and altered oral hygiene [79]. Behavioural modification including preventative dental screening, a healthy diet, avoiding frequent snacking and limiting the frequency of sugar consumption can effectively prevent dental caries during pregnancy [80, 81].

In regard to unfavourable beliefs about oral hygiene, in three studies, participants believed that tooth brushing should be ‘avoided’ during and after pregnancy [12, 29, 39]. In one of these studies, participants stated family influence or fear of brushing being harmful to their teeth or babies as the reasons for such belief [12]. This finding contradicts the evidence that encourages regular oral hygiene practices during and after pregnancy [79]. However, tooth brushing should be avoided after vomiting as it increases the risk of tooth erosion [82]. Nevertheless, both personal and professional preventative modalities such as toothbrushing, dental flossing, using mouthwash, and regular dental visit and checkups are considered safe during pregnancy [79].

Our review identified several environmental and individual contributing factors in relation to oral health-related unfavourable beliefs and dental service utilization during pregnancy. Economic status was shown to be positively associated with higher knowledge scores [50, 56] and negatively associated with the belief that dental treatment is not safe during pregnancy [31]. Similarly, in many studies, higher education level was found to be a protective predictor of low oral health knowledge [28, 33, 49, 50, 56]. Other predictors of better maternal oral health knowledge include having children [28, 49], older age [49], and routine dental visits prior to pregnancy [31]. Race and ethnicity were examined in a few studies; however, no significant association between oral health knowledge with education, ethnicity, and level of employment was reported in at least one study [54]. These inconsistent findings can be explained by the variety of questions the authors of different studies asked the participants for assessing oral health knowledge and the inconsistency among studies in categorizing a specific statement as knowledge or attitude.

### Implications of oral health-related unfavourable beliefs on prenatal care

Unfavourable beliefs about oral health can have consequences such as avoidance of dental treatment and dental service utilization [10]. Dinas et al. found that women who considered dental treatment to be unsafe during pregnancy were less likely to visit a dentist [31]. Unfavourable beliefs may also contribute to oral health conditions which can be due to dental care avoidance or improper oral hygiene [58, 83]. In a study by Jain et al. [12], a significant positive correlation was observed between having dental conditions (conditions were not specified by the authors) and beliefs pertaining to avoiding dental visits during pregnancy. Delayed dental care-seeking behaviour can lead to the progression of dental conditions and complications such as odontogenic abscess, facial cellulitis, severe pain, and tooth loss [80, 84]. These complications can adversely impact pregnancy outcomes and the overall well-being of pregnant women including nutrition, sleep time, and mental health [66, 85].

### Clinical implications

Our review identified that oral health literacy was directly associated with presumed myths and unfavourable beliefs [86]. Therefore, it is important to engage pregnant women in delivering evidence-based oral health education [87]. Pregnancy is the best time to bring about positive behavioural change in women [88]. A study by Erchick et al. [89] concluded that pregnant women were more likely to adopt their oral hygiene intervention as it has a direct impact on the health of the fetus and newborn. Although no studies have examined the association between the level of oral health knowledge and the source of such knowledge during pregnancy, some studies reported that culture, elder family members and peers from participants’ networks, including friends, are the origins of such beliefs [12, 22, 23]. Surprisingly, healthcare professionals have also been reported as a source of misinformation regarding oral health [90, 91]. This finding highlights the importance of integration of primary oral healthcare knowledge and prevention in the primary healthcare model. A study by George et al. [92] in Australia engaged midwives in delivering oral health education to pregnant women. Similarly, Ragade et al. [93] and Adeniyi et al. [94] actively engaged primary healthcare workers in preventative oral health interventions in the United States and Canada, respectively. Integration of oral health education within the primary care delivery model can significantly help to reduce and dismantle commonly held misbeliefs during pregnancy [92]. The wide range of existing unfavourable beliefs regarding oral health during pregnancy emphasizes the importance of integrating preventative and primary oral healthcare services in routine prenatal care. Furthermore, it underscores the importance of enhancing the oral health expertise of primary healthcare practitioners and disseminating this knowledge to expectant mothers through oral health educational interventions during prenatal care sessions. These interventions should be focused on the individualized and risk based oral health needs of pregnant women and their soon to be born babies.

### Limitations of the included studies

Several limitations were identified in the reviewed studies. In most of the studies, authors did not distinguish between knowledge and attitude which resulted in using these two concepts interchangeably. Further, a quarter of the included studies did not elaborate on the process of developing their survey questionnaires and the sampling method was not reported in almost one-third of the studies. Only seven studies had a random sampling method which ensured the representativeness of the sample while a majority of the studies incorporated a convenience sampling method which limits the external validity of the findings. Further, recruiting the subjects from a specific clinic or hospital, either public or private, resulted in a homogenous sample.

### Limitations of the review

Although this study is the first of its kind to attempt a systematic review of commonly held oral health beliefs during pregnancy and to discuss the contributing factors and consequences, the findings should be considered in light of the limitations. Since our review included both qualitative and quantitative studies, we were unable to separate our findings based on the study design. Some beliefs were only reported by a single individual in a qualitative study henceforth the generalizability of these misbeliefs is not guaranteed. The prevalence of the beliefs was not compared according to the country or region; however, this objective did not seem feasible given the large variety of reported beliefs. Further studies could assess differences in beliefs among pregnant women from various cultures and with different socioeconomic statuses. Furthermore, this review limited the population to pregnant women and new mothers; unfavourable beliefs among healthcare providers could be systematically explored in future studies to see if any implementation for improving the oral health knowledge of these groups is required.

## Conclusions

This systematic review revealed that unfavourable beliefs regarding oral health during pregnancy and dental care still exist and are held in many geographic locations. Oral health and dental care unfavourable beliefs may result from a lack of information and oral health awareness by pregnant women and their primary care providers. Pregnant women actively seek information related to their baby’s health and are at risk of obtaining wrong information from different sources such as media and social networks. Healthcare providers should ensure their own, and their patients’ knowledge of oral health and dental care during pregnancy is up to date. Avoiding dental treatment due to misinformation and unfavourable beliefs can lead to untreated dental conditions related to adverse pregnancy outcomes such as preeclampsia, preterm birth and low birth weight. Therefore, preventative oral healthcare services and education should be integrated into the primary care model during pregnancy.

### Supplementary Information

**Additional file 1: Table S1.** PRISMA checklist.

**Additional file 2: Table S2.** Search Strategy.

**Additional file 3: Figure S1.** Risk of bias assessment for each included study using the CLARITY tool.

**Additional file 4: Table S3.** Original statements and their prevalence in each included study.

## Data Availability

All data generated or analysed during this study are included in this published article [and its supplementary information files].

## Abbreviations

RoB: Risk of Bias
PRISMA: Preferred Reporting Items for Systematic Reviews and Meta-Analysis
PROSPERO: International Prospective Register of Systematic Reviews
