# Supplementary material for: Unfavourable beliefs about oral health and safety of dental care during pregnancy: a systematic review
Source: BMC Oral Health. 2023 Oct 15;23:762. doi: 10.1186/s12903-023-03439-4 (PMC10577919; doi:10.1186/s12903-023-03439-4)
Supplement: Supplementary file 2 — Additional file 2: Table S2. Search Strategy. [file 12903_2023_3439_MOESM2_ESM.pdf]

Table S2. Search Strategy.

	Database			
Concept	PubMed	CINAHL	Scopus	MEDLINE(Ovid)
Belief	“beliefs”[All Fields] OR “culture”[MeSH Terms] OR “culture”[All Fields] OR “belief”[All Fields] OR “beliefs”[All Fields] OR “percept”[All Fields] OR “perceptibility”[All Fields] OR “perceptible”[All Fields] OR “perception”[MeSH Terms] OR “perception”[All Fields] OR “perceptions”[All Fields] OR “perceptional”[All Fields] OR “perceptive”[All Fields] OR “perceptiveness”[All Fields] OR “percepts”[All Fields] OR “view eijing”[Journal] OR “view”[All Fields] OR “attitude”[MeSH Terms] OR “attitude”[All Fields] OR “attitudes”[All Fields] OR “attitude s”[All Fields] OR “knowledge”[MeSH Terms] OR “knowledge”[All Fields] OR “knowledge s”[All Fields] OR “knowledgeability”[All Fields] OR “knowledgeable”[All Fields] OR “knowledgeably”[All Fields] OR “knowledges”[All Fields] OR “myth”[All Fields]	(MH "Health Beliefs") OR "belief*" OR "*perception" OR "view" OR "attitude*" OR (MH "Attitude") OR (MH "Knowledge") OR "knowledge" OR (MH "Health Knowledge") OR "myth*" OR (MH "Folklore")	TITLE-ABS-KEY (belief* OR *perception OR knowledge OR view* OR attitude* OR myth*))	belief.mp. or Culture/OR view.mp. OR *perception/ OR attitude*.mp. OR Knowledge/ or Health Knowledge, Attitudes, Practice/ or knowledge.mp.OR myth*.mp.
Population	“gravidity”[MeSH Terms] OR “gravidity”[All Fields] OR “pregnant”[All Fields] OR “pregnants”[All Fields] OR (“pregnancy”[MeSH Terms] OR “pregnancy”[All Fields] OR “pregnancies”[All Fields] OR “pregnancy s”[All Fields]) OR ((“expect”[All Fields] OR “expectable”[All Fields] OR “expectance”[All Fields] OR “expectant”[All Fields] OR “expectative”[All Fields] OR “expected”[All Fields] OR “expecting”[All Fields] OR “expects”[All Fields] OR “motivation”[MeSH Terms] OR “motivation”[All Fields] OR “expectancies”[All Fields] OR “expectancy”[All Fields] OR “expectation”[All Fields] OR “expectations”[All Fields]) AND (“mother s”[All Fields] OR “mothered”[All Fields] OR “mothers”[MeSH Terms] OR “mothers”[All Fields] OR “mother”[All Fields] OR “mothering”[All Fields])) OR (“pregnant women”[MeSH Terms] OR (“pregnant”[All Fields] AND “women”[All Fields]) OR “pregnant women”[All Fields]) OR (“pregnant women”[MeSH Terms] OR (“pregnant”[All Fields] AND “women”[All Fields]) OR “pregnant women”[All Fields] OR (“pregnant”[All Fields] AND “woman”[All Fields]) OR “pregnant woman”[All Fields])	"pregnant*" OR (MH "Expectant Mothers") OR (MH "Pregnancy") OR "pregnancy" OR "pregnant wom#n" OR (MH "Expectant Mothers")	TITLE-ABS-KEY (pregnan* OR "pregnant women" OR "pregnant woman" OR "expect* mother*")	pregnan*.mp. OR pregnant women.mp. or Pregnant Women/OR pregnant woman.mp. or Pregnant Women/OR expect* mother*.mp.
Oral/dental health	“oral health”[MeSH Terms] OR (“oral”[All Fields] AND “health”[All Fields]) OR “oral health”[All Fields] OR (“oral health”[MeSH Terms] OR (“oral”[All Fields] AND “health”[All Fields]) OR “oral health”[All Fields] OR (“dental”[All Fields] AND “health”[All Fields]) OR “dental health”[All Fields]) OR ((“mouth”[MeSH Terms] OR “mouth”[All Fields] OR “oral”[All Fields]) AND “care”[All Fields]) OR (“dental care”[MeSH Terms] OR (“dental”[All Fields] AND “care”[All Fields]) OR “dental care”[All Fields])	(MH "Oral Health") OR "oral health" OR (MH "Dental Health Services") OR "dental health" OR "oral care" OR (MH "Dental Care") OR "dental care"	TITLE-ABS-KEY ("oral health" OR "dentall health" OR "dental care" OR "oral care")	Oral Hygiene/ or oral care.mp. OR dental care.mp. or Dental Care/ OR oral health.mp. or Oral Health/ OR dental health.mp.
Linking concepts	1 AND 2 AND 3<br>N=3429	1 AND 2 AND 3<br>N=195	1 AND 2 AND 3<br>N=622	1 AND 2 AND 3<br>N=430
