# Supplementary figures and images for: Unfavourable beliefs about oral health and safety of dental care during pregnancy: a systematic review
Source: BMC Oral Health. 2023 Oct 15;23:762. doi: 10.1186/s12903-023-03439-4 (PMC10577919; doi:10.1186/s12903-023-03439-4)

**Figure S1.** Risk of bias assessment for each included study using the CLARITY tool.

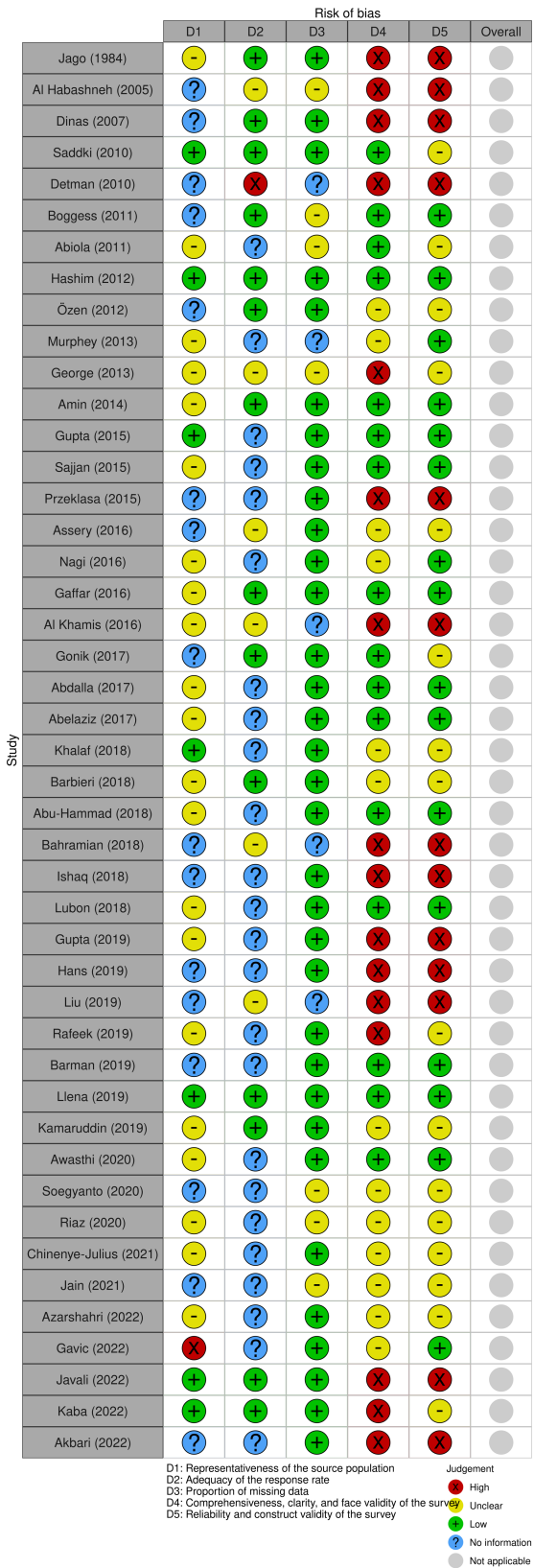

Supplement: Supplementary file 3 — Additional file 3: Figure S1. Risk of bias assessment for each included study using the CLARITY tool. [file 12903_2023_3439_MOESM3_ESM.pdf]
