# Supplementary material for: Unfavourable beliefs about oral health and safety of dental care during pregnancy: a systematic review
Source: BMC Oral Health. 2023 Oct 15;23:762. doi: 10.1186/s12903-023-03439-4 (PMC10577919; doi:10.1186/s12903-023-03439-4)
Supplement: Supplementary file 4 — Additional file 4: Table S3. Original statements and their prevalence in each included study. [file 12903_2023_3439_MOESM4_ESM.pdf]

Table S3. Original statements and their prevalence in each included study.

Author (year)	Statement (n (%))	Author (year)	Statement (n (%))	Author (year)	Statement (n (%))
**Jago et al. (1984)**	- Pregnancy itself causes tooth decay bad (109 (25%))<br>- Pregnancy itself causes gum disease (27 (6%))<br>- A tooth is lost in each pregnancy (35 (8%))	**Assery (2016)**	- Pregnancy and lactation cause tooth decay (28 (11%))<br>- Pregnancy leads to tooth decay (125 (50%))<br>- The fetus absorbs calcium from the mother's teeth (93 (37%))<br>- Pregnancy can cause inflammatory gum: (90 (36%))<br>- Dental treatment during pregnancy adversely affects the fetus (128 (51%)): X-rays (127 (50%)), anesthetic injection (77 (31%)), dental fillings (40 (16%)), tooth extraction (15 (6%)), and calculus removal (4 (2%))	**Liu et al. (2019)**	- Dental visits should be avoided during pregnancy<br>- Toothache is normal during pregnancy<br>- The baby absorbs the mother's calcium<br>- Calcium supplements suffice for dental problems prevention<br>- Uncomfortable feeling in teeth is because of "yeet hay" (internal heat)<br>- Pregnant women should avoid taking medicine<br>- Dental check-ups may lead to bleeding and bacterial infection in the baby<br>- The environment, noise, and materials of dental clinics are harmful to the baby
**Al Habashneh et al. (2005)**	- Pregnant women should not visit a dentist during pregnancy - a barrier to dental visits (71 (11%))<br>- A tooth is lost in each pregnancy (116 (19%))<br>- Calcium is absorbed from the mother's teeth by the fetus (294 (47%))	**Nagi et al. (2016)**	- Certain medications can affect the fetus's growth (146 (33%))<br>- Exposure to radiation during pregnancy is hazardous (149 (33%))<br>- Pregnancy is a cause of teeth loss (112 (25%))	**Rafeek et al. (2019)**	- Dental care should be avoided during pregnancy unless it is an emergency (60 (37%))<br>- Dental treatment during pregnancy is not safe - a barrier to dental visits (74 (46%))
**Dinas et al. (2007)**	- Dental treatment is not safe during pregnancy - a dental visit barrier (83 (19%))<br>- Dental treatment may have a negative effect on pregnancy outcomes (307 (72%))	**Gaffar et al. (2016)**	- Dental treatment during pregnancy negatively affects fetal health (53 (27%))<br>- Dental visits cannot be scheduled during pregnancy (91 (46%))	**Barman et al. (2019)**	- Dental treatment has negative effects on the fetus - a dental visit barrier (0% of Savar women and 100.0% of Soara women) *
**Saddki et al. (2010)**	- Dental treatment is not safe during pregnancy - a dental visit barrier (14 (16%))	**Al Khamis et al. (2016)**	- It is normal to have dental pain during pregnancy<br>- Pregnant women lose a tooth in each pregnancy<br>- The fetus absorbs the calcium from the mother's teeth<br>- Dental treatment during pregnancy may harm the baby	**Llena et al. (2019)**	- Dental radiography during pregnancy is absolutely contraindicated (123 (88%))<br>- Dental anesthesia is contraindicated during pregnancy (116 (83%))
**Detman et al. (2010)**	- Pain medication is not prescribed for pregnant women<br>- Dental anesthetics are not used for pregnant women<br>- Dentists do not provide treatment for pregnant women<br>- Teeth should not be extracted during pregnancy<br>- The fetus absorbs the calcium from the mother's teeth	**Gonik et al. (2017)**	- Tooth decay is normal during pregnancy (urban: 37 (18%), suburban: 11 (20%))<br>- Tooth loss is normal during pregnancy (urban: 17 (8%), suburban: 5 (9%))<br>- Bleeding gum is normal during pregnancy (urban: 94 (45%), suburban: 31 (57%))<br>- Swollen gum is normal during pregnancy (urban: 37 (18%), suburban: 27 (50%))<br>- Toothache is normal during pregnancy (urban: 62 (30%), suburban: 5 (9%))<br>- Frequency (%) of oral and dental practices	**Kamaruddin et al. (2019)**	- Dental treatment during pregnancy is not safe - a dental visit barrier (20 (26%))<br>- Pregnant women should avoid dental treatment unless it is an emergency (44 (58%))

			which were considered safe in urban and suburban centres: tooth cleaning (164 (79%), 49 (91%)), tooth x-ray (21 (10%), 11 (20%)), filling cavities (35 (17%), 25 (46%)), fluoride treatment (19 (9%), 11 (20%)), local anesthesia (10 (5%), 9 (17%)), Antibiotic prescription (33 (16%), 14 (26%)), Pain medication prescription (29 (14%), 6 (11%)).		
**Boggess et al. (2011)**	- It is not safe for pregnant women to get routine dental care such as checkups and cleanings (76 (13%))<br>- A woman can lose a tooth just because she is pregnant (112 (19%))	**Abdalla et al. (2017)**	- Pregnancy causes tooth loss (227 (76%))<br>- Pregnancy causes calcium loss (261 (87%))	**Awasthi et al. (2020)**	- Pregnancy is one of the causes of gum problems (28 (25%))<br>- It is not necessary to visit a dentist during pregnancy (41 (36%))<br>- Pregnancy causes tooth loss (0 (0%)
**Abiola et al. (2011)**	- Pregnancy is a cause of gum problems (67 (15%))<br>- Dental visits are unnecessary during pregnancy (106 (23%))	**Abelaziz et al. (2017)**	- Changes that occur to the mouth and teeth during pregnancy are normal (248 (93%))<br>- If one is not exposed to oral/dental problems during pregnancy, they do not need dental checkups (80 (30%))<br>- Providing medical procedures to treat oral/dental problems during pregnancy can affect your baby (112 (42%))<br>- Treatment of oral/dental problems should be delayed til after delivery (63 (24%))	**Soegyanto et al. (2020)**	- Dentists cannot provide any dental treatment during pregnancy - a barrier to dental visit (3 (2%))<br>- A tooth is lost in each pregnancy (12 (7%))<br>- Calcium is absorbed from the mother’s teeth by the fetus (68 (41%))
**Hashim (2012)**	- A tooth is lost in each pregnancy (333 (44%))	**Khalaf et al. (2018)**	- The fetus is responsible for tooth loss during pregnancy as they absorb calcium from their mothers (277 (76%))<br>- It is possible to lose teeth because of pregnancy (298 (82%))<br>- It is unsafe for the pregnant women to visit the dentist even for regular checkups (232 (64%))	**Riaz et al. (2020)**	- Pregnant women should seek dental treatment only in case of emergency (192 (74 %))
**Özen et al. (2012)**	- Dentists cannot provide any dental treatment during pregnancy - a dental visit barrier (34 (10%))<br>- A tooth is lost in each pregnancy (151 (43%))<br>- Calcium is absorbed from the mother’s teeth by the fetus (256 (73%))	**Barbieri et al. (2018)**	- The increase in dental caries occurrences is due to weakening of teeth by loss of calcium (57 (29%))<br>- The increase in dental caries occurrences is due to hormonal changes (37 (19%))<br>- Dental treatment should be avoided during the entire pregnancy (18 (9%))<br>- Dental treatment should only be in case of emergency (19 (10%))	**Chinenye-Julius et al. (2021)**	- Visit a dentist is harmful to the fetus (137 (36%))<br>- Pregnant women do not need to visit dental clinics (157 (41%))
**Murphey (2013)**	- The mother's diet during pregnancy affects the child's teeth color<br>- Routine dental procedures would predispose mothers to premature birth or harm the unborn baby<br>- Dental radiographs harm the baby	**Abu-Hammad et al. (2018)**	- Dental radiography is not safe during pregnancy (36 (10%))<br>- Pregnant women can lose teeth only because of pregnancy (201 (55%))<br>- Visiting a dentist during pregnancy for check-up and scaling is not safe (218 (61%))	**Jain et al. (2021)**	- One should not brush during pregnancy (32 (8%))<br>- One should wait for a few days to few weeks before starting brushing after delivery (241 (63%))<br>- One should avoid going to the dentist during pregnancy even in case of toothache (56 (15%))<br>- Gum bleeding during pregnancy is common and

	- Noises made by dental equipment frighten or harm the baby<br>- Calcium is absorbed from the mother's teeth during pregnancy which can be compensated through swishing and keeping the milk in the mouth before swallowing				needs no special attention (65 (17%))<br>- Oral health during pregnancy has nothing to do with the baby's health (21 (6%))<br>- Injection for numbness for dental treatment during pregnancy affects the development of the baby's organs (93 (24%))<br>- Dental extraction during pregnancy causes miscarriage (83 (22%))
**George et al. (2013)**	- Dental treatment is not safe during pregnancy - a dental visit barrier (77 (32%))<br>- Dental treatment should be avoided during pregnancy unless it is an emergency (63 (26%))	**Bahramian et al. (2018)**	- One does not need to visit a dentist during pregnancy if they take calcium supplements<br>- At least one tooth is destructed in each pregnancy<br>- Calcium from the mother's teeth is consumed for formation of fetus skeleton<br>- Medication prescribed by dentists may harm the fetus<br>- Radiography causes miscarriage<br>- Dental anesthetics cause miscarriage	**Azarshahri et al. (2022)**	- It is normal to lose a tooth during pregnancy (346 (56%))<br>- Calcium is absorbed from pregnant women's teeth by the fetus (168 (27%))<br>- Dental treatment could harm unborn child - reasons for not using dental services (15 (6%))<br>- Poor oral health is normal during pregnancy - reasons for not using dental services (1 (<1%))
**Amin et al. (2014)**	- It is normal to lose a tooth during pregnancy (60 (14%))<br>- Calcium is absorbed from a pregnant woman's teeth by the fetus (248 (59%))<br>- Dental treatment could harm unborn child - reasons for not using dental services (33 (15%))<br>- Poor oral health is normal during pregnancy - reasons for not using dental services (4 (2%))	**Ishaq et al. (2018)**	- Dental treatments are not safe during pregnancy (94 (78%))<br>- Dental extraction cannot be done during pregnancy (101 (83%))<br>- X-rays are not safe during pregnancy (105 (87%))	**Gavic et al. (2022)**	- Local anesthesia is not safe for pregnant women (145 (45%))<br>- Attitudes about safety of treatments during pregnancy: Routine teeth cleaning (320 (98%)), production of fillings or crowns (72 (21%)), periodontal treatment (99 (30%)), extractions (31 (10%)), intraoral/extraoral radiograms (5 (2%))
**Gupta et al. (2015)**	- Pain in gums or bleeding gums is normal during pregnancy (13 (7%))<br>- Visiting a dentist during pregnancy is not safe (46 (23%))<br>- Pregnancy is a cause of loosening teeth (34 (17%)<br>- After delivery teeth shouldn't be brushed (82 (41%))<br>- Treatment of dental related problems during pregnancy is not safe (84 (42%))	**Lubon et al. (2018)**	- Poor oral hygiene causes cavities that enter the abdomen and negatively affect the baby	**Javali et al. (2022)**	- X-rays affect mother and baby (412 (93%))<br>- Medications affect mother and baby (431 (97%))<br>- Pregnant need dental checkups (432 (97%))
**Sajjan et al. (2015)**	- Exposure to radiation is hazardous to the baby (106 (32%))<br>- Certain medications affect the development of the child (133 (40%))	**Gupta et al. (2019)**	- Dental treatment during pregnancy is not safe - a dental visit barrier (8 (16%))	**Kaba et al. (2022)**	- Pregnant women should not visit a dentist for dental treatments (173 (56%))<br>- Dental treatment during pregnancy is harmful to the baby (159 (52%))<br>- Pregnancy causes tooth loss (264 (86%))<br>- It is normal to have bleeding gums during pregnancy (264 (86%))<br>- Scaling during pregnancy is not safe (165 (54%))<br>- Pregnant women cannot have dental extraction (264

					(86%))<br>- Dental X-ray during pregnancy is harmful to the baby (227 (74%))<br>- Pregnant women can lose a tooth only because of pregnancy (289 (94%))<br>- Visiting the dentist during pregnancy for a check-up is not safe (63 (21%))
**Przeklasa et al. (2015)**	- Dental anesthetics cannot be administered during pregnancy (60 (45%))<br>- Teeth may not be treated during pregnancy (11 (8%))<br>- Dental visit is not necessary during pregnancy (25 (19%))	**Hans et al. (2019)**	- Pain in gums and bleeding are normal during pregnancy (144 (64%))<br>- Pregnant females should avoid dental treatment unless it is an emergency (202 (90%))<br>- Pregnancy causes loosening of the teeth (181 (80%))<br>- It is not safe to visit a dentist during pregnancy (156 (69%))	**Akbari et al. (2022)**	- Pregnant women's teeth decay easily due to calcium absorption by the fetus (562 (70%))<br>- Calcium supplement consumption during pregnancy stimulates the eruption of primary teeth (547 (68%))<br>- Receiving an anesthetic injection for dental treatment during pregnancy affects the fetus's organs (398 (50%))<br>- Tooth extraction during pregnancy causes miscarriage (230 (29%))<br>- Bleeding gums during pregnancy is common and does not need any specific treatment (229 (29%)<br>- A pregnant woman should not visit a dentist during her pregnancy regardless of having any oral problems (51 (6%))<br>- A woman should delay brushing her teeth for a few days or weeks after delivery (22 (3%))

* The exact number of pregnant women in each tribe is not provided by the authors.
